# Detection and phylogenetic analysis of a novel tick-borne virus in *Haemaphysalis longicornis* ticks and sheep from Shandong, China

**DOI:** 10.1186/s12985-021-01704-y

**Published:** 2021-11-27

**Authors:** Lijun Shao, Ruiheng Chang, Lin Liu, Yongjin Wang, Yun Gao, Shaoqing Wang, Hengyi Sun, Guoyu Niu

**Affiliations:** 1 School of Public Health, WeiFang Medical University, Weifang, 261053 China; 2 Tianjin International Joint Academy of Biomedicine, Tianjin, 300457 China; 3 Immune-Path Biotechnology (Suzhou) Co., Ltd, Suzhou, 215000 China

**Keywords:** Dabieshan tick virus, Ticks, Sheep, Shandong Province

## Abstract

Dabieshan tick virus (DTV) was first identified in *Haemaphysalis longicornis* from Hubei Province, China in 2015. However, its pathogenic potential to animals and human remains to be further explored. In this study, a total of 170 engorged ticks and 22 sheep serum samples were collected from Taian and Yantai city, Shandong Province to investigate the presence of DTV. The results of qRT-PCR revealed the positive rate of 13.6% (3/22) in sheep serum and 8.2% (14/170) in attached ticks, respectively. Phylogenetic analysis demonstrated a close evolutionary relationship among those DTV isolates from animal and ticks, and DTV might be relatively conservative in evolution. These findings are the first to demonstrate molecular evidence of DTV in domestic animals. Nonetheless, whether or not causing disease in animals, DTV deserves further investigation.

## Background

Ticks are recognized as the second largest group of ectoparasites after mosquito that are exclusively specialized obligate hematophages [1]. Ticks are the main arthropod vectors of pathogens including protozoa, bacteria and viruses with hosts ranging from wildlife to domestic animals and human worldwide [2]. Recently, much more attention has been paid to emerging diseases caused by tick-borne viruses (TBVs) because of their significant impact on domestic animals and human health [3]. TBVs include a variety of viruses from different genera, among which the genus *Phlebovirus* is the most important and usually named tick-borne phleboviruses (TBPVs). TBPVs contain several important emerging viruses that are pathogenic to human, such as severe fever with thrombocytopenia syndrome virus (SFTSV) and Heartland virus (HRTV) [4, 5].

Dabieshan tick virus (DTV) is also one emerging TBPV and was first identified in *Haemaphysalis longicornis* from Hubei Province, China in 2015 [6]. It is reported that DTV was an intermediate species with very close evolutionary relationships to several TBVs, such as Yongjia tick virus 1 and Uukuniemi virus [7, 8]. Previous studies have found that DTV was widely distributed in ticks from Shandong Province and Zhejiang Province, and there was no significant difference in nucleotide sequence between those isolates. In addition, the carrying rate of DTV in ticks from Zhejiang Province was much higher than Shandong Province [8]. However, less is known about the arthropod vector ecology, host range and animal pathogenicity of DTV.

In this study, sequence analysis of DTV in ticks and sheep sera sampled from DTV-endemic areas of Shandong Province was performed to further understand the circulation of DTV in China. Through sequence gathering, evolution matrix construction, and evolutionarily related analysis, we came to understand the genomic characterization as well as the evolutionary relationship among different virus strains. Our findings suggested domestic animals were likely to play an important role in the spread of DTV as replication or preservation hosts.

## Methods

### Sample collection and RNA extraction

**Fig. 1:**
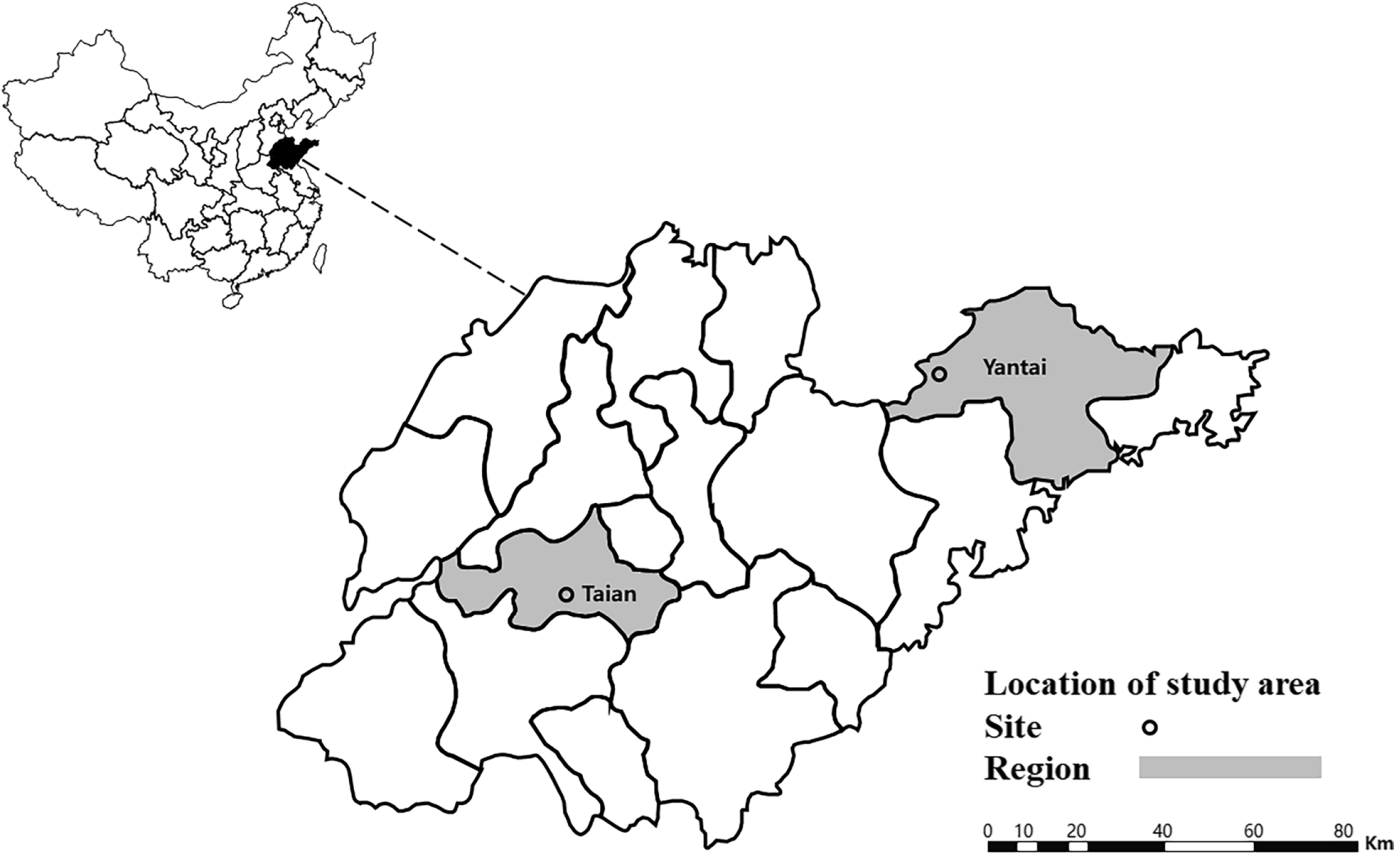
Location of Shandong Province in China (left), and location of Taian city and Yantai city within the province where tick samples were collected in 2019

Samples were collected in Taian (116°91′E, 36°19'N) and Yantai (120°03′E, 37°15′N), Shandong Province, China (Fig. 1). Sheep was the most common domestic animal species in these regions and adult sheep raised in free range were selected in this study. Approximately 3 mL of blood samples were collected from sheep jugular veins and stored at 4 °C for one day, then sera were obtained after centrifugation According to the actual situation, 3–15 engorged ticks were collected from the body surface of each sheep with tweezers and stored in a cool and ventilated place for two weeks. After identified morphologically by an experienced field biologist from Chinese Academy of Inspection and Quarantine, individual tick was transferred into liquid nitrogen and then homogenized in 500 μL of chilled DMEM using a tissue homogenizer (Qiagen, Germany). After centrifuged at 4 °C and 10,000 g for 5 min, the clarified supernatant of tick homogenates and sera were prepared for RNA extraction using TIANamp RNA extraction kit (Tiangen, China) following the manufacturer’s instructions.

### PCR for detection of DTV

The detection of DTV was performed by qRT-PCR targeting the S segment, with DTV-F (TGC TCC TCT CCG CAC ACC T), DTV-R (TGG CAA GTA GAG GAA ACT GGT GA) and DTV-P (FAM-TCC CTC CAG CCA TCA CCA CCT CC-BHQ1) described before [7]. All qRT-PCR-positive samples were further confirmed by nested PCR using primer sets Out-F (GGC AGC ACT TTC ACG GAT G), Out-R (CCC CTG TCA TGT CTA ATC AAT GG), In-F (GCA AGC AGA GCC TCA AGA AGC), In-R (GCC AGA TTG CGA TCC AAG TAT G). Amplified products were visualized by SYBR® Safe (Thermo, USA) after 1% agarose gel electrophoresis.

### Bioinformatics analysis

SeqMan and MegAlign of DNASTAR software package (Lasergene, USA) were utilized to edit and align the obtained sequences. MEGA5.1 software was used for phylogenetic analysis by constructing the neighbor-joining evolutionary tree with 1000 replicates, compared with previous published viral sequences of DTV strains. The relevant GenBank accession numbers range from MZ965029 to MZ965036.

### Ethical clearance

All animal experiments were carried out in strict accordance with the guidelines of the Animal Committee of Weifang Medical University. This study was reviewed and approved by the Ethic Committee of Weifang Medical University. The collection of animal blood was permitted by Daolang District and Laizhou County Forestry Bureau.

## Results and discussion

### Sample collection

From May through September 2019, 170 engorged ticks were collected from 22 sheep in Shandong Province, of which 89 ticks from 12 sheep in Taian city, and 81 ticks from 10 sheep in Yantai city (Table 1). All ticks were identified as *Haemaphysalis longicornis* and stored in a cool and ventilated place to digest the blood in their body. Then, each tick or serum sample was grouped for viral RNA detection assays.

**Table 1:** Detection of DTV viral RNA in sheep and ticks collected from Shandong, China

	Sheep			Attached ticks		
	Sample ID	qRT-PCR	Nested-PCR	No	qRT-PCR	Nested-PCR
Taian	TADL-01	−	/	12	−	/
	TADL-02	−	/	9	−	/
	TADL-03	+	+	5	4	2
	TADL-04	−	/	3	−	/
	TADL-05	−	/	5	−	/
	TADL-06	−	/	7	−	/
	TADL-07	−	/	7	−	/
	TADL-08	−	/	6	−	/
	TADL-09	−	/	9	2	1
	TADL-10	−	/	15	2	1
	TADL-11	+	−	3	1	−
	TADL-12	−	/	8	−	/
Total		2	1	89	9	4
Yantai	LZSS-01	−	/	11	−	/
	LZSS-02	−	/	14	−	/
	LZSS-03	−	/	7	1	1
	LZSS-04	−	/	8	−	/
	LZSS-05	−	/	8	−	/
	LZSS-06	**+ **	+	4	3	1
	LZSS-07	−	/	7	−	−
	LZSS-08	−	/	9	1	−
	LZSS-09	−	/	7	−	/
	LZSS-10	−	/	6	−	/
Total		1	1	81	5	2

“/” indicates that no experimental operation has been carried out

### Detection of DTV RNA in ticks and sheep

According to the result of qRT-PCR, DTV RNA was detected in 14 ticks (out of 170, 8.2%) and 3 sera samples (out of 22, 13.6%) in this study. 9 ticks (out of 89, 10.1%), 2 sera samples (out of 12, 16.7%) from Taian, and 5 ticks (out of 81, 6.2%), 1 sera sample (out of 10, 10%) from Yantai, respectively (Table 1). This result indicated a high prevalence of DTV in engorged ticks and domestic animals, compared to that of unfed ticks (0.67%) in the same region [7]. It seemed that sheep could enhance virus replication or play a positive role in virus spread. Besides, we also found that DTV RNA was detected in sheep from different areas, and the prevalence was also high, maintaining at about 10%.

### Phylogenetic analysis

**Fig. 2:**
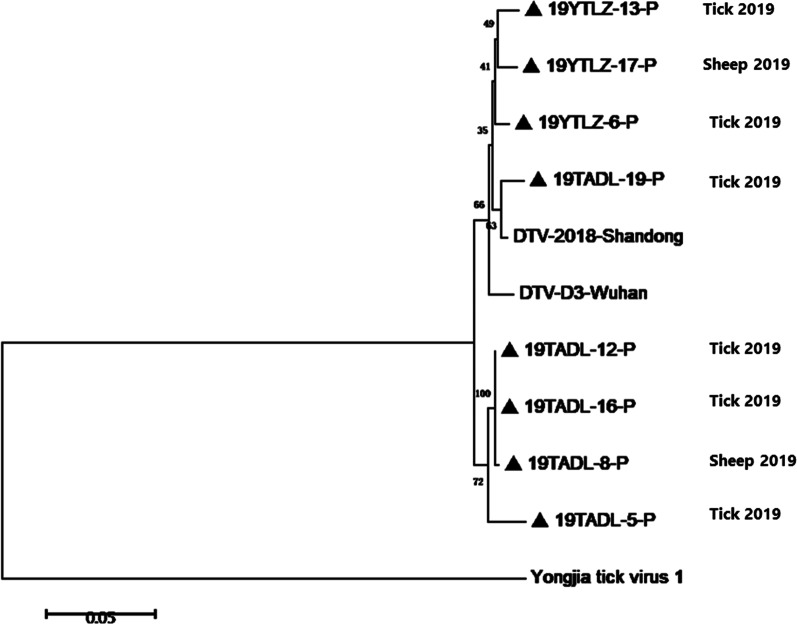
Phylogenetic analysis of DTV S-segment sequences amplified from sheep and tick samples. A phylogenetic tree based on S-segment sequences (548 bp) by the NJ method using MEGA 5.1 is shown. DTV-D3-Wuhan indicates the tick-derived sequence amplified from Hubei Province in 2015. DTV-2018-Shandong indicates the tick-derived sequence amplified from Shandong Province in 2018

According to the latest taxonomic information from the International Committee on Taxonomy of Viruses (ICTV) in 2019, DTV is classified as a new member of Uukuvirus genus, Phenuiviridae [9]. Recent studies have demonstrated that DTV was closely related with Yongjia tick virus, Uukuniemi virus and Okutama tick virus in phylogeny and its pathogenicity to animals and human remains unclear due to the lack of further research [7, 8]. In this study, the partial S segment of DTV was amplified and sequenced from 8 of 17 qRT-PCR-positive samples and all the sequences obtained from sheep and ticks had a 95.1–99.8% nucleotide identity with each other. Pairwise distances analysis demonstrated a close evolutionary relationship among those DTV isolates from animal and ticks in these epidemic regions. Interestingly, the isolate 19YTLZ-13-P from tick shared nearly the same identity (98.2%) with 19YTLZ-17-P from attached sheep (LZSS-006). Meanwhile, similar results occurred in another group from Taian, the isolates 19TADL-12-P and 19TADL-16-P from ticks grouped more closely with19TADL-8-P from attached sheep (TADL-003) (Fig. 2). These suggested a potential link of DTV infection between sheep and ticks, and DTV might circulate in the endemic areas among ticks and many kinds of other domestic animals, such as cattle, pigs, dogs, etc. Besides, these 8 DTV isolates obtained in this study were closely related to those found in Hubei Province, indicating that DTV from different regions had high similarity and DTV might be relatively conservative in evolution, which was also consistent with our previous research results.

In addition to transmitted by ingesting the blood of infected hosts, arboviruses have been proved to spread from infected ticks to other ticks attaching the same even seropositive host by co-feeding [10, 11]. Under experimental conditions, the transmission of SFTSV from infected to uninfected *Haemaphysalis longicornis* occurred by co-feeding in BALB/c mice and the author speculated that this transmission pathway was of great significance to the survival of TBV in nature [12]. In the present study, 22 groups of corresponding RNA samples were obtained from ticks and sheep serum samples, and DTV was detected simultaneously in sheep serum and corresponding ticks among 3 groups, which suggested that the potential viremia might promote the flow and transmission of virus in ticks and animal hosts. Nevertheless, not all ticks from DTV RNA positive sheep were detected carrying DTV, this perhaps because of too few copies of this virus under the detection threshold of qRT-PCR resulting from insufficient blood intake. Otherwise, DTV was detected positive in some ticks of 4 groups of samples, but negative in corresponding sheep blood and these discordant results suggest that ticks carrying DTV had not yet caused high level of viremia in host animals owing to some factors, such as insufficient blood exchange.

Infectious virus, a competent insert vector, and susceptible reverse hosts are the three basic elements for the stable establishment of a viral growth cycle [13]. It has been proven that threshold viremia level of 10^2.0–4.7^ 50% lethal dose/mL is sufficient for some TBVs infections, like Colorado tick fever virus, Russian spring–summer encephalitis virus, and louping ill virus [14–16]. In this study, DTV was detected in domestic sheep, although the copy numbers of viral RNA were low (approximately 6.0 × 10^4^ copies/mL, 95% CI = 5.6–7 × 10^4^ copies/mL). The partial S segment of DTV was successfully amplified and the results confirmed the existence of DTV in domestic sheep. However, only two isolates were obtained from all serum samples, which indicated that most infected sheep might have either a short period or low level of viremia.

## Conclusions

Our data provides epidemiological evidences for DTV infection in ticks and sheep in endemic areas and suggests that *Haemaphysalis longicornis* might serve as a vector for the transmission of DTV to sheep. Whether or not causing disease in sheep and other animals, DTV warrants further investigation.

## Data Availability

Access to raw data can be acquired by contacting the corresponding author via email.

## Abbreviations

DTV: Dabieshan tick virus
TBVs: Tick-borne viruses
TBPVs: Tick-borne phleboviruses
SFTSV: Severe fever with thrombocytopenia syndrome virus
HRTV: Heartland virus
ICTV: International Committee on Taxonomy of Viruses
